# Taste Receptor Cells Generate Oscillating Receptor Potentials by Activating G Protein-Coupled Taste Receptors

**DOI:** 10.3389/fphys.2022.883372

**Published:** 2022-05-25

**Authors:** Yoshiki Nakao, Katsumi Tateno, Yoshitaka Ohtubo

**Affiliations:** Graduate School of Life Science and Systems Engineering, Kyushu Institute of Technology, Kitakyushu, Japan

**Keywords:** oscillation, receptor potentials, action potentials, *in situ* perforated patch clamping, fungiform taste bud cells

## Abstract

The receptor potentials of taste receptor cells remain unclear. Here, we demonstrate that taste receptor cells generate oscillating depolarization (*n* = 7) with action potentials in response to sweet, bitter, umami, and salty taste substances. At a lower concentration of taste substances, taste receptor cells exhibited oscillations in membrane potentials with a low frequency and small magnitude of depolarization. Although the respective waves contained no or 1–2 action potentials, the taste receptor cells generated action potentials continuously in the presence of taste stimuli. Both the frequency and magnitude of oscillations increased when the concentration was increased, to 0.67–1.43 Hz (*n* = 3) and Δ39–53 mV (*n* = 3) in magnitude from −64.7 ± 4.2 to −18.7 ± 5.9 mV, which may activate the ATP-permeable ion channels. In contrast, a sour tastant (10-mM HCl) induced membrane depolarization (Δ19.4 ± 9.5 mV, *n* = 4) with action potentials in type III taste receptor cells. Interestingly, NaCl (1 M) taste stimuli induced oscillation (*n* = 2) or depolarization (Δ10.5 ± 5.7 mV at the tonic component, *n* = 9). Our results indicate that the frequency and magnitude of oscillations increased with increasing taste substance concentrations. These parameters may contribute to the expression of taste “thickness.”

## Introduction

Taste sensation for five basic tastes (sweet, bitter, umami, sour, and salty) is initiated by the interaction between taste substances and taste receptor cells in the mouth. Taste receptor cells express G protein-coupled or ionotropic taste receptors on their apical membranes, which are exposed to a harsh environment, such as drastic changes in osmotic pressure or pH. In contrast, their basolateral membranes are present in stable physiological conditions. Hence, the maintenance of their physiological environment is essential to investigate the signal transduction mechanisms of taste. A previous study used *in situ* whole-cell patch clamping in which taste substances were applied only to the apical membranes. This study indicated that mouse taste receptor cells generated depolarizing receptor potentials with action potentials in response to NaCl and HCl (Ohtubo et al., 2001), which activate ionotropic taste receptors (Zhang et al., 2019; Heck et al., 1984). Although taste receptor cells generate action currents that are mediated by the sweet, bitter, and umami G protein-coupled taste receptors (Furue and Yoshii, 1997; Yoshida et al., 2006; Noguchi et al., 2003), the receptor potential changes were unknown because the cell-attached or extracellular loose-seal recordings were used to prevent the washout of cytosolic components. Furthermore, as the activation of G protein-coupled taste receptors induces intracellular Ca^2+^ concentration changes (Dando and Roper, 2009; Huang and Roper, 2010; Roebber et al., 2019), Ca^2+^ imaging was chosen for investigating the taste signal transduction. Therefore, despite the existence of several studies on taste responses at taste receptor cells, the receptor potentials mediated by G protein-coupled taste receptors remain unclear.

A single taste bud contains 10–100 taste bud cells (TBCs). Elongated TBCs are classified into three cell types (type I, II, and III) based on their morphology and functions (Murray, 1973; Chaudhari and Roper, 2010; Roper, 2013). Of the average 42 TBCs of a single fungiform taste bud of a mouse (Ohtubo and Yoshii, 2011), type I cells are the most abundant and are believed to function as glia-like supporting cells (Lawton et al., 2000; Bartel et al., 2006). Type II cells account for approximately 25% of a taste bud (Ohtubo and Yoshii, 2011) and function as taste receptor cells by expressing different G protein-coupled receptors for sweet, umami, and bitter taste substances (Nelson et al., 2002; Nelson et al., 2001; Chandrashekar et al., 2000; Adler et al., 2000). Type III cells comprise approximately 5% of a taste bud (Ohtubo and Yoshii, 2011) and are sensitive to acid (Huang et al., 2008; Kataoka et al., 2008). Hence, approximately 30% of TBCs are taste receptor cells (type II and type III cells). As individual taste receptor cells encode individual taste qualities (Adler et al., 2000; Nelson et al., 2001), it is difficult to obtain stable recordings in response to all five tastes from a given taste receptor cell in a single taste bud. It is likely that these essential features of TBCs contribute to the difficulty in obtaining electrophysiological recordings from taste receptor cells in response to taste substances that activate G protein-coupled taste receptors.

**FIGURE 1:**
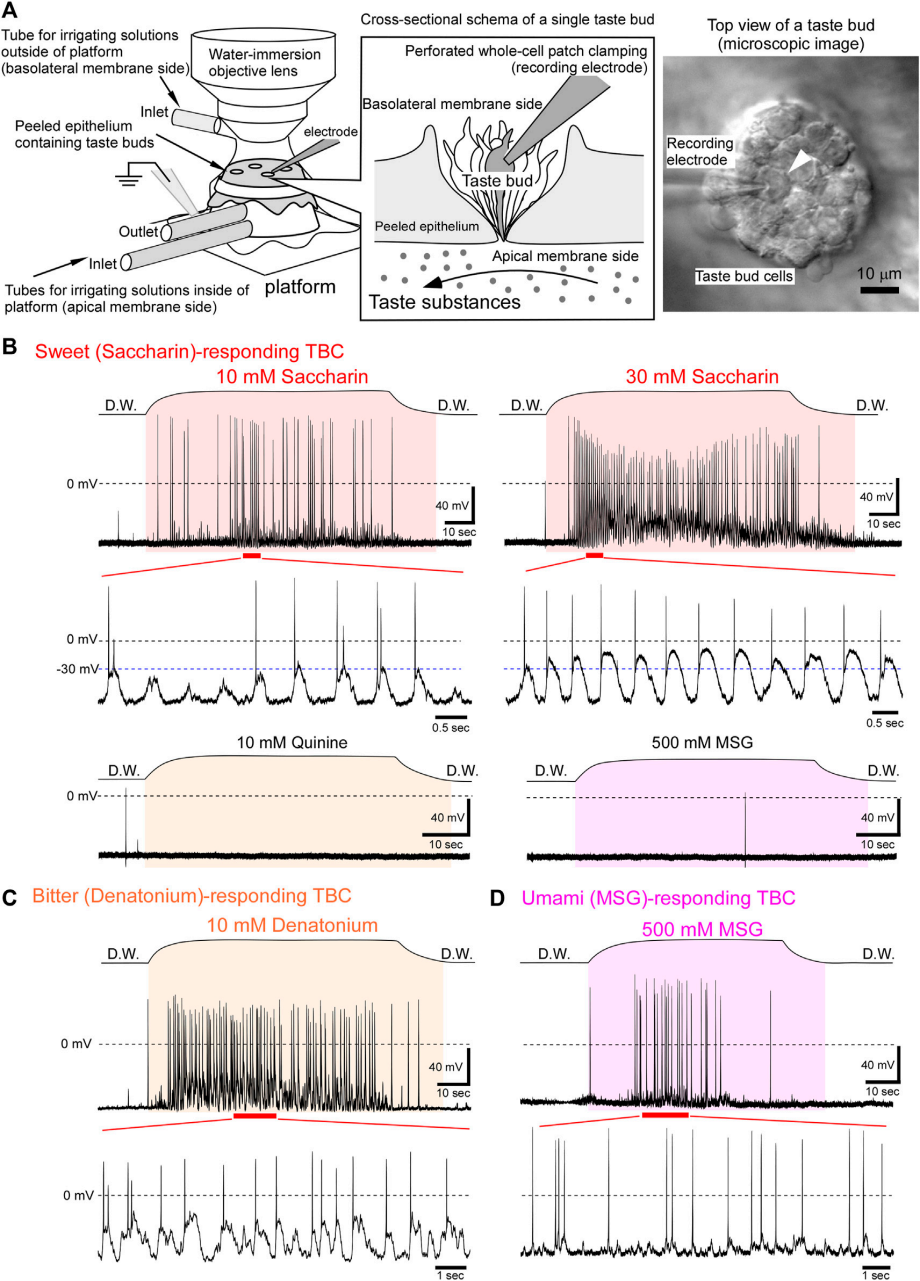
Oscillating receptor potentials to sweet, bitter, or umami taste substances. **(A)** Experimental setup for performing *in situ* perforated whole-cell recording from fungiform TBCs. Each taste substance dissolved in deionized water was applied only to the apical membranes through tubes connected to the platform. A differential interference contrast image of the basolateral membrane side of TBCs (right image). Under visual guidance, the recording electrode was placed on a single TBC (arrowhead), and then the perforated whole-cell recording was performed. **(B)** A sweet taste substance, saccharin, generated oscillating depolarizations with action potentials. This TBC responded to saccharin in a concentration dependent manner, although it did not respond to 10 mM quinine and 500 mM MSG. **(C)** A TBC responded to a bitter substance and generated oscillations with firings. **(D)** An umami tastant induced oscillating receptor potentials with firings. The period between the beginning and end of taste stimulation is indicated in the colored fill patterns. D.W, deionized water.

In the present study, we performed an “*in situ* perforated whole-cell patch clamping” to TBCs in the peeled lingual epithelium to elucidate the receptor potential changes mediated by G protein-coupled taste receptors (Figure 1A). Using this system, it was possible to apply the taste substances only to the apical membranes of TBCs (Furue and Yoshii, 1997; Ohtubo et al., 2001) and to keep the intracellular small molecules, such as second messengers, inside the TBCs. Our results indicate that taste receptor cells generate oscillating depolarization with action potentials in response to sweet, bitter, umami, and salty taste substances. The advantages of oscillating depolarization for taste signal transduction are discussed.

## Materials and Methods

All experimental protocols were conducted in compliance with the Guiding Principles for the Care and Use of Animals in the Field of Physiological Sciences approved by the Council of the Physiological Society of Japan, the Animal Institutional Review Board of Kyushu Institute of Technology, and the guidelines of the U.S. National Institutes of Health. The protocols were approved by the president of Kyushu Institute of Technology.

### Preparation of Peeled Lingual Epithelium

We peeled the mouse lingual epithelium containing the fungiform papillae as described previously (Furue and Yoshii, 1998; Ohtubo et al., 2001). Briefly, we euthanized 5–7-week-old dd Y-strain male mice, anesthetized using CO_2_ *via* decapitation, cut the tongue, hypodermically injected a collagenase solution into the tongue, and incubated the tongue at 25°C for 3–4 min. We then peeled off the epithelium using forceps under a stereomicroscope. The peeled epithelia were then mounted on a recording platform, with the basolateral membrane side of the taste receptor cells facing upward.

The recoding platform was placed under a microscope (50BX; Olympus Corporation, Tokyo, Japan), equipped with a ×60 water-immersion objective. The apical (inside the recoding platform) and basolateral (outside the recoding platform) membranes of the TBCs were irrigated continuously and separately using deionized water and physiological saline, respectively. Taste substances dissolved in deionized water were applied only to the apical membranes of the TBCs.

### *In situ* Perforated Whole-Cell Recordings

Membrane potentials and voltage-gated currents of taste receptor cells were assessed under *in situ* perforated whole-cell current- or voltage-clamp conditions. Recording electrodes filled with an intracellular electrode solution containing amphotericin B (Fujifilm Wako Pure Chemical Corporation, Osaka, Japan) and Pluronic® F-127 (Sigma-Aldrich, MO, United States) were attached to the basolateral membranes of single TBCs when observed under a microscope. The membrane voltages or voltage-gated currents in the respective modes were amplified and filtered at 10 kHz using a voltage-clamp amplifier (Axopatch 200B; Axon Instruments, CA, United States). Data were digitized using an A/D converter (Digidata 1322A; Axon Instruments) and then stored using the pCLAMP data acquisition and analysis software (ver. 9.0; Axon Instruments).

Amphotericin B stock solution (25–50 mg/ml) was prepared by diluting amphotericin B with dimethylsulfoxide (DMSO), followed by trituration using micropipette and subsequent sonication. This stock solution was stored at −20°C and used within 5 days. Pluronic® F-127 solution contained 20 mg/ml Pluronic® F-127 dissolved in the K-gluconate solution, which was subjected to sonication. The intracellular electrode solution was prepared in the K-gluconate solution by adding amphotericin B stock solution to 300–500 μg/ml final concentration and Pluronic® F-127 solution to 250 μg/ml final concentration. After this, the intracellular electrode solution was sonicated in a light-shielding glass bottle for 1–3 min and filtered using a 0.22 μm membrane-filter unit (MILLEX®-GP; Merck, Darmstadt, Germany). The intracellular electrode solution was kept on ice, protected from the light, and renewed every 4 h.

Although amphotericin B has low permeability to Ca^2+^ ions, if there is a large difference in the Ca^2+^ concentrations between the cytosol and intracellular electrode solutions, this may affect the cytosolic Ca^2+^ concentration. In our experimental condition, the free Ca^2+^ concentration in the intracellular electrode solution was approximately 0.1 μM, which was estimated using the Ca-EGTA calculator v1.3 with the constants from Theo Schoenmakers’ Chelator (https://somapp.ucdmc.ucdavis.edu/pharmacology/bers/maxchelator/CaEGTA-TS.htm). The influx of free Ca^2+^ ions *via* amphotericin B may have a lesser effect on the cytosolic free Ca^2+^ concentration, although the exact concentration of free Ca^2+^ in the cytosol is unknown.

The recording electrodes were pulled using a micropipette puller (PC-10; Narishige, Tokyo, Japan) and the tip was fire polished. The resistance of the recording electrode filled with the intracellular electrode solution ranged from 2 to 4 MΩ in physiological saline. After the formation of the GΩ seal, the membrane-capacitance changes were monitored by continuously applying rectangular pulse from −70 to −65 mV. As amphotericin B diffuses to the tip of the recording electrode, it gradually increases the electrode–cell conductance. Therefore, capacitive currents loaded by a voltage-step increase with increasing electrical communication between TBCs and the recording electrode. The membrane capacitance increased slowly over time and reached the steady-state levels at approximately 10–20 min. The readings for a family of voltage-gated currents under voltage-clamp mode were recorded after series resistance and membrane-capacitance compensations. Further, the mode was changed to current-clamp mode. The membrane potentials of TBCs were controlled between −70 and −60 mV by injecting steady currents. Under this condition, the membrane potentials of TBCs in response to respective taste substances that were applied only to the apical membrane were recorded. We neglected the liquid junction potential of approximately −10 mV.

### Taste Stimuli Only to the Apical Membranes of Taste Bud Cells

Taste stimuli to the apical membrane and the irrigation to the basolateral membranes of TBCs were performed as described in previous studies (Ohtubo et al., 2001; Furue and Yoshii, 1998). Briefly, the apical membranes of TBCs were adapted by irrigating deionized water using the tubes that were connected to the inside of the platform (Figure 1A). The basolateral membranes were continuously irrigated using physiological saline *via* a tube located near the water-immersed objective. After the readings for the family of voltage-gated currents were recorded, respective taste substances dissolved in the deionized water were irrigated under the current-clamp mode. Although the apical membrane (inside the recording platform) was continuously irrigated with deionized water, the readings for the family of voltage-gated currents could be recorded from the basolateral membrane of the TBCs, indicating that the measurement system exhibits minimal leakage of solution from the apical membrane side to the basolateral membrane side and vice versa, as described in previous studies (Ohtubo et al., 2001; Furue and Yoshii, 1998).

**FIGURE 2:**
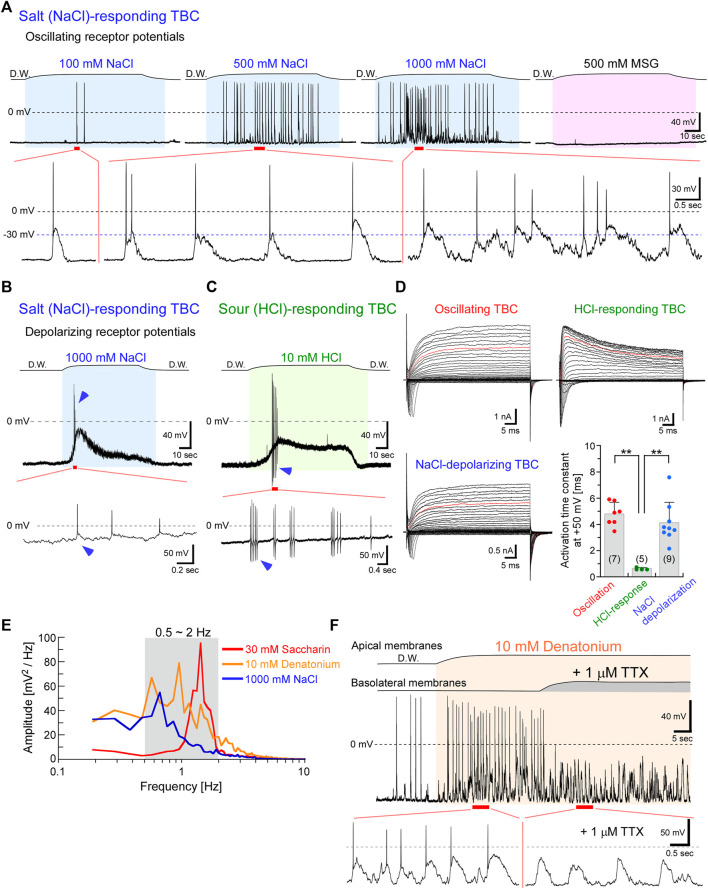
NaCl taste stimuli induced oscillating or depolarizing receptor potentials, whereas HCl stimuli generated depolarizations in type III cells. **(A)** Representative oscillations responded to a series of NaCl concentrations. The frequency and magnitude of oscillations increased with increasing concentration. However, this TBC did not respond to MSG. **(B)** Another TBC indicated depolarization with action potentials (arrowheads) in response to 1 M NaCl. **(C)** Sour stimuli (10 mM HCl) also induced depolarization with action potentials and exhibited after-hyperpolarization (arrowheads). **(D)** A family of voltage-gated currents. The activation kinetics of the outward rectifying currents at +50 mV (red traces in each family current) was significantly faster in TBCs responding to HCl than in others (**p < 0.01; one-way analysis of variance followed by Scheffe’s multiple comparison test). Data are presented as means ± standard deviations. The numerals in the parentheses represent the number of TBCs examined. **(E)** Power spectral densities of the oscillations. **(F)** The application of 1 μM tetrodotoxin (TTX) to the basolateral membranes during the response to bitter taste substances inhibited the generation of action potentials but did not affect the oscillations. The response to bitter taste substances in the presence of TTX was obtained from the same TBC indicated in Figure 1C. The period between the beginning and end of taste stimulation is indicated in the colored fill patterns. D.W, deionized water.

In some experiments, after the recording, the solution exchange rate for the apical membrane side was measured by the changes in the liquid-junction potentials between the recording electrode solution and solutions inside the platform. The recording electrode was placed at the taste pore after removal of the taste bud in which the TBC was investigated. Subsequently, the solution inside the platform (apical membrane side) was changed from deionized water to 1 M NaCl. The time constants of onset and washout obtained were 7.34 ± 0.60 s (*n* = 9) and 9.31 ± 0.69 s (*n* = 9), respectively. The solution exchange rate of the apical membranes is shown above the respective taste responses in Figures 1, 2. The period between the beginning and end of the stimulation is indicated in the colored fill patterns.

### Activation Kinetics of Outwardly Rectifying Currents

The activation time constant of outwardly rectifying currents at +50 mV was obtained using a single exponential curve fitting to the current trace using the following equation: I (t) = A{1 − exp [(−t + d)/τ]}; where I (t) is the flow of current at time (t), A is the current amplitude at infinite time, d is the delay required to obtain an adequate fit of a single exponential function, and τ is the time constant. The activation time constant of the outwardly rectifying currents at +50 mV obtained from seven oscillating-receptor-potential TBCs, five HCl-depolarization TBCs, and nine NaCl-depolarization TBCs was analyzed using one-way analysis of variance followed by Scheffe’s multiple comparison test. *p* < 0.05 was considered statistically significant.

### Analysis of Oscillation Frequency and Magnitude

The frequency of oscillation was analyzed using the Clampfit data analysis software (ver. 11.2.1.00; Axon Instruments). A low pass filter with a cut-off frequency of 15 Hz was applied to remove spikes, and then baseline adjustment was performed by subtracting the average over the entire trace. Fast Fourier Transformation using a rectangular window was performed every 524,288 points (=10.48576 s), and the spectra of the entire trace were obtained using 50% window overlap to determine the spectral mean. The DC component was not included in the spectra.

The magnitude of oscillation was calculated by subtracting the lower voltage value from the upper voltage value in each wave after the taste substance reached the apical membrane. The average upper and lower voltage values obtained from five waves in respective response traces to tastants were used for the calculation.

### Solutions

All solutions were prepared using deionized water. Physiological saline (pH 7.4/NaOH) comprised 150 mM NaCl, 5 mM KCl, 2 mM CaCl_2_, 0.5 mM MgCl_2_, 10 mM glucose, and 5 mM Hepes (Dojindo Laboratories, Kumamoto, Japan). The collagenase solution contained 4 mg/ml collagenase type I (Fujifilm Wako Pure Chemical Corporation) dissolved in physiological saline. The K-gluconate solution (pH 7.2/KOH) comprised 120 mM K-gluconate, 2.4 mM CaCl_2_, 5 mM MgCl_2_, 10 mM EGTA, 30 mM KOH, 0.3 mM Na_3_GTP, 5 mM Na_2_ATP, and 10 mM Hepes. We used the following five basic taste stimuli: saccharin sodium dihydrate as a sweet substance, denatonium benzoate and quinine hydrochloride dihydrate as bitter substances, monosodium glutamate (MSG) as an umami substance, sodium chloride (NaCl) as a salty substance, and hydrochloric acid (HCl) as a sour substance. Taste substances were purchased from Fujifilm Wako Pure Chemical Corporation.

## Results

Of the 88 TBCs, 21 responded to one taste substance among the five basic taste substances investigated (Table 1). When the irrigating solution on the apical membrane was changed from deionized water to a sweet (i.e., saccharin-containing) solution, the membrane potential of the TBCs was depolarized with or without action potentials and then repolarized to the resting level despite the presence of saccharin (Figure 1B). This change was repeatedly observed during sweet stimulation of the apical membrane. Such oscillatory changes in membrane potentials are defined as “oscillation” in this study, although the magnitude of the oscillation varied among different tastants.

**TABLE 1:** Taste-responding TBC profiles.

Cell no.	Sac	Den	QHCl	MSG	NaCl	HCl
1	Osci	ND	**−**	**−**	ND	ND
2	Osci	ND	ND	ND	ND	ND
3	**−**	Osci	ND	ND	ND	ND
4	**−**	**−**	ND	Osci	ND	ND
5	**−**	**−**	ND	Osci	ND	ND
6	**−**	**−**	**−**	**−**	Osci	ND
7	**−**	**−**	ND	**−**	Osci	ND
8	**−**	**−**	**−**	**−**	Depo	ND
9	**−**	**−**	ND	**−**	Depo	ND
10	**−**	**−**	ND	**−**	Depo	ND
11	**−**	**−**	ND	**−**	Depo	ND
12	**−**	**−**	ND	**−**	Depo	ND
13	ND	ND	ND	ND	Depo	ND
14	**−**	**−**	ND	ND	Depo	ND
15	**−**	**−**	ND	**−**	Depo	**−**
16	**−**	**−**	ND	ND	Depo	ND
17	ND	ND	ND	**−**	ND	Depo
18	ND	ND	ND	ND	ND	Depo (20 mM)
19	**−**	**−**	ND	**−**	**−**	Depo
20	**−**	**−**	**−**	**−**	**−**	Depo
21	ND	ND	ND	ND	ND	Depo

Taste-responding TBC profiles induced by taste substances listed in column heading. Den, 10 mM denatonium; Depo, depolarization; “ − ”, no response; HCl, 10 or 20 mM HCl; MSG, 500 mM monosodium glutamate; NaCl, 1 M NaCl; ND, not determined; Osci, oscillating depolarization; Sac, 30 mM saccharin; QHCl, 10 mM quinine HCl, Each taste substance dissolved in deionized water was applied only to apical membranes of TBCs.

Saccharin (10 mM) induced oscillating depolarizations with action potentials (Figure 1B). The rising phase of the respective waves comprised no or 1–2 action potentials. The firing frequency increased qualitatively with increasing concentrations of saccharin. Further, the peak magnitude of oscillations was increased from approximately −30 to −10 mV with the increasing concentration (Figure 1B). These oscillating changes in response to saccharin were obtained from 2 of 75 TBCs examined. Similar oscillating receptor potential changes were observed in response to the bitter taste substances (Figure 1C, 10 mM denatonium, 1 of 67 TBCs) and umami taste substance (Figure 1D, 500 mM MSG, 2 of 55 TBCs). Because G protein-coupled taste receptors for sweet, bitter, and umami taste substances are expressed in type II cells (Adler et al., 2000; Chandrashekar et al., 2000; Nelson et al., 2001; Nelson et al., 2002), our results suggest that the type II taste receptor cells generate the oscillating receptor potentials with action potentials.

Interestingly, TBCs responding to 1 M NaCl exhibited two types of receptor potentials, oscillation and depolarization (Figures 2A,B; Table 1). The magnitude and frequency of oscillation were increased with increasing NaCl concentrations (Figure 2A). Oscillating changes responsive to 1 M NaCl were obtained from 2 of 37 TBCs examined. In contrast to the oscillating depolarization, 9 of the 37 TBCs showed membrane depolarization with a phasic component and a subsequent tonic component (Figure 2B). The membrane potential of TBCs started to depolarize when the taste substance reached the apical membrane, and it continuously depolarized during the presence of the stimuli (and did not repolarize while the stimuli were present); repolarization to the resting membrane potential was observed when the tastant was washed out. In this study, “depolarization” refers to the membrane potentials maintaining depolarization in the presence of the taste stimuli. The magnitude of depolarization with the tonic component in TBCs responding to 1 M NaCl was increased to Δ10.5 ± 5.7 mV (*n* = 9) from the resting membrane potential of approximately −60 mV.

A sour taste substance (HCl) induced depolarization with action potentials (Figure 2C, 5 of 27 TBCs). The magnitude of depolarization in TBCs responding to 10 mM HCl was Δ19.4 ± 9.5 mV (*n* = 4). Although both NaCl and HCl taste stimuli generate action potentials at the rising phase of depolarization, the after-hyperpolarization was observed in the HCl-responding TBCs, but not in the NaCl-responding TBCs (arrowheads in Figures 2B,C). Previous studies reported that the action potentials in type III cells displayed after-hyperpolarization in response to current-injection–induced membrane depolarization, whereas those in type II cells did not (Kimura et al., 2014; Ohtubo, 2021). Therefore, the cell types may differ between NaCl- and HCl-responding TBCs.

As the kinetics differ between type II and type III cells (Kimura et al., 2014; Iwamoto et al., 2020; Takeuchi et al., 2021), we compared the activation kinetics of the outward rectifying currents that were recorded before the application of the taste substance, to identify the cell types of HCl-responding TBCs. Type II cells mainly expressed Cs^+^-insensitive and slowly activated outwardly rectifying currents that passed through voltage-gated hemichannels and calcium homeostasis modulator 1 and 3 (CALHM1/3) channels and generated tail currents during repolarization. In contrast, Type III cells expressed Cs^+^-sensitive and rapidly activated K^+^ currents but did not generate tail currents. Type I cells expressed relatively small Cs^+^-insensitive outwardly rectifying, small tailed, and relatively small voltage-gated Na^+^ currents. The cell types of the HCl-responding TBCs were identified based on the electrophysiological features of the voltage-gated currents.

The activation kinetics of voltage-activated outward currents at +50 mV obtained from TBCs responding to HCl was 0.63 ± 0.07 ms (*n* = 5), which was significantly faster than that obtained from oscillating TBCs (4.80 ± 0.89 ms, *n* = 7, *p* < 0.0001, Figure 2D) and NaCl-depolarizing TBCs (4.13 ± 1.56 ms, *n* = 9, *p* = 0.0002). The activation kinetics of TBCs responding to HCl was similar to that obtained from immunohistochemically identified type III cells (0.86 ± 0.41 ms, *n* = 6) (Takeuchi et al., 2021). These data suggest that HCl-responding TBCs are type III cells, whereas NaCl-depolarizing TBCs are not type III cells. As type II cells generate relatively larger voltage-gated Na^+^ and outwardly rectifying currents (Figure 2D) than type I cells (Kimura et al., 2014; Takeuchi et al., 2021), the NaCl-depolarizing TBCs are likely to be type II cells.

The frequency analysis showed that the peak of the oscillations was 0.67–1.43 Hz (*n* = 3) in response to 30 mM saccharin (peak value = 1.43 Hz), 10 mM denatonium (0.95 Hz), and 1 M NaCl (0.67 Hz; Figure 2E). This oscillation at approximately 1 Hz frequency is suitable for the effective recovery from the inactivation of the voltage-gated Na^+^ channels because the slow time constant of the recovery was approximately 0.8 s at −70 mV in type II taste receptor cells of mice (Ohtubo, 2021). Therefore, type II taste receptor cells can continuously generate action potentials in response to taste substances (Figures 1, 2).

The magnitude of oscillation was Δ39–53 mV during the changes (*n* = 3) in response to 30 mM saccharin (upper value, −12 mV; lower value, −65 mV; Δ53 mV), 10 mM denatonium (−23 mV; −69 mV; Δ46 mV), and 1 M NaCl (−22 mV; −61 mV; Δ39 mV). Additionally, when applied to the basolateral membrane, tetrodotoxin (TTX) inhibited the generation of action potentials but did not affect the oscillations (Figure 2F). The magnitude of oscillation was similar in the presence and absence of TTX (Figure 2F), suggesting that TTX-sensitive Na^+^ channels are not involved in the generation of oscillating depolarization.

## Discussion

In the present study, we demonstrated that taste receptor cells generate oscillating receptor potentials with action potentials in response to sweet, bitter, umami, and salty taste substances using “*in situ* perforated whole-cell patch clamping.” A previous study using a similar method and mouse soft palate taste buds showed that the restricted application of 10 mM quinine hydrochloride (a bitter substance) to the apical membrane elicited oscillatory receptor potentials with action potentials on their upstrokes (Noguchi, 2003). Although our sample size was only seven cells that elicited oscillating receptor potentials, our results reconfirmed that a bitter substance can induce oscillation and newly demonstrated that sweet, umami, and salty taste substances also induce oscillating receptor potentials. Additionally, we showed that the frequency and magnitude of oscillations increased with increasing taste concentrations. These oscillating membrane potential changes that occur in a concentration-dependent manner may affect the secreting patterns of neurotransmitters, such as ATP, because ATP is a transmitter that conducts signal transduction between taste receptor cells and taste nerves (Finger et al., 2005).

Taste signal conduction from type II cells to taste nerves occurs *via* both action potential-dependent and action potential-independent ATP secretion through CALHM1/3 channels (Huang and Roper, 2010; Murata et al., 2010). CALHM1/3 channels are nonselective, voltage- and/or Ca^2+^-sensitive large pore-forming channels (Taruno et al., 2013; Ma et al., 2018) that are activated by the membrane depolarization of > −30 mV (Iwamoto et al., 2020). Previous studies have shown that these channels can open without deactivation during a long depolarization period under voltage-clamp conditions (Kimura et al., 2014; Takeuchi et al., 2021). Therefore, the depolarizing oscillation of > −30 mV may itself activate CALHM1/3 channels, which could be possible in case of action potential-independent ATP secretion. Additionally, the prolonged opening of CALHM1/3 channels may disrupt cellular homeostasis because ions and small molecules, such as ATP, move through CALHM1/3 channels according to electrochemical gradients. Thus, oscillating receptor potentials are suitable for the maintenance of physiological homeostasis of type II taste receptor cells by limiting the opening of ATP-permeable channels.

Because of the slow recovery from the inactivation of voltage-gated Na^+^ channels in mouse TBCs, TBCs hardly generate high-frequency firings in response to membrane depolarization induced by current injection (Ohtubo, 2021). Meanwhile, TBCs continuously generate action currents (action potentials) in response to sweet, bitter, and umami taste substances during taste stimuli (Furue and Yoshii, 1997; Noguchi et al., 2003; Yoshida et al., 2006). This discrepancy can be explained by considering membrane potential oscillations. During these oscillations, recovery from the inactivation of voltage-gated Na^+^ channels accelerated by the repolarization of membrane potentials enables repetitive firing during taste stimuli in the taste receptor cells (Figures 1, 2). This repetitive firing may contribute to the action potential-dependent ATP secretion.

Analysis of the activation kinetics of outward rectifying currents suggested that HCl-responding cells were type III cells, which is in agreement with the results of the previous studies that type III cells sense sour taste substances (Huang et al., 2008; Kataoka et al., 2008). Additionally, our data suggested that NaCl-depolarizing TBCs were type II cells because type II cells generated relatively larger voltage-gated Na^+^ and outwardly rectifying currents than type I cells (Kimura et al., 2014; Iwamoto et al., 2020). Similar results were observed in a recent study on sodium taste. According to that study, CALHM1/3 expressing taste cells can generate a depolarization driving action potential by activating the amiloride-sensitive epithelial sodium channel (Nomura et al., 2020). Furthermore, a high salt concentration recruited two aversive taste pathways that activate the bitter- and sour-taste-sensing cells (Oka et al., 2013). Bitter-sensing type II cells may generate oscillating depolarization in response to 1 M NaCl as bitter substance (10 mM denatonium) generated oscillation (Figure 1C). Further studies are needed to clarify the relationships between salty taste transduction mechanisms, including two aversive taste pathways and the two types of receptor potential changes, oscillation and depolarization.

Overall, type II taste receptor cells generate oscillating depolarization in response to sweet, bitter, and umami tastants that activate G protein-coupled receptors. Additionally, the frequency and magnitude of oscillations were increased in a concentration-dependent manner. These results suggest that the information on taste “thickness” can be converted into both firing frequency and magnitude of oscillations. Further studies are needed to elucidate the relationship between the ATP-secretion patterns and receptor potential changes, besides elucidation of molecular mechanisms that generate the oscillations.

## Data Availability

The original contributions presented in the study are included in the article/supplementary material, further inquiries can be directed to the corresponding author.
